# The Role of Surgery to Relieve Symptomatic Cutaneous T-Cell Lymphoma Refractory to Medical Treatments

**DOI:** 10.1155/2024/6645278

**Published:** 2024-04-26

**Authors:** Wesley Q. Zhang, Emily E. Hecox, Shireen Dogar, Marc E. Walker

**Affiliations:** ^1^School of Medicine, University of Mississippi Medical Center, Jackson, Mississippi 39216, USA; ^2^Division of Plastic Surgery, Department of Surgery, University of Mississippi Medical Center, Jackson, Mississippi 39216, USA

## Abstract

**Background:** Though mycosis fungoides (MF) is the most common type of cutaneous T-cell lymphoma (CTCL), it has no curative treatment. The aim of current topical and systemic treatment is centered around relieving symptoms and optimizing disease-free time. The use of surgical management to achieve the same goals of symptomatic reduction is not well described in the current literature.

**Methods:** We present a case of refractory MF that failed chemotherapy, radiotherapy, and UV light therapy. Despite medical management, the tumor burden progressed to significant compression neuropathy of the ulnar and median nerves.

**Results:** To reduce tumor burden and attempt to provide symptomatic relief, a surgical plan was developed to include radical resection of the tumor of the left upper extremity (LUE) with release of the cubital tunnel, carpal tunnel, Guyon canal, and coverage with split-thickness skin graft. The patient reported decreased symptomatology interfering with her daily activities and, overall, a better quality of life postoperatively.

**Conclusion:** Surgical intervention, in addition to established medical standards of care, for symptomatic relief of compression neuropathy from tumor mass effect for refractory CTCL should be considered to achieve quality of life goals for patients.

## 1. Introduction

Cutaneous T-cell lymphomas (CTCLs) are a rare group of non-Hodgkin lymphomas distinguished by the proliferation of T lymphocytes that have a worldwide incidence of 6.4 cases per million individuals per year [1]. The most common type of CTCL is mycosis fungoides (MF) which often presents with tumors, patches, and plaques on the skin. In the United States, MF is more commonly found in Black Americans and in males [2, 3]. Clinically, this disease usually progresses over years to decades and often starts with macules on the skin that change to plaques and finally to tumors [4]. The standard of treatment for CTCL involves medical management with both skin-directed therapies for patients with early-stage disease and systemic therapies with late-stage/refractory disease.

Currently, no treatments are considered curative for MF, and most patients suffer from relapse [5]. Most existing treatments, which include chemotherapy, radiotherapy, and UV phototherapy, are chosen based on disease stage and ability to improve overall quality of life [6]. However, the surgical management of MF is not well-described in the literature, even though it has the potential to align with some of the same goals of care as existing standards of care. Herein, we present a case of MF that was surgically managed to improve quality of life. Case presentation, clinical features, and treatment approach will be reviewed.

## 2. Case

A 31-year-old Black female diagnosed with MF in 2016 was referred to our clinic complaining of paresthesia of the left upper extremity (LUE). Nodules first began to grow across her body including her upper extremities, lower extremities, and lips in 2012. In 2016, she was diagnosed with MF at MD Anderson and was determined to be at T3 N1-3M0 B1 on TNMB staging (at least stage IIB) with evidence of lymph node involvement but without visceral involvement. The dermatologist and hematologist at MD Anderson had different recommendations for her treatment, with the former preferring monotherapy chemotherapy and the latter preferring CHOP + pralatrexate or MTX. Because her staging was IIB and because of the risk of CHOP causing rebound flares, the patient and her home medical team (at the University of Mississippi Medical Center) decided to proceed with MTX only. She was not trialed on CHOP-R or SCT after that. While she was at MD Anderson, she was also referred to the stem cell center for a consultation, but she had cancelled her appointment for an unknown reason. Despite treatment with methotrexate and other drugs such as Istodax (a histone deacetylase inhibitor), Targretin, and topical steroids, her condition worsened, especially the nodules on her upper lip. For the lesions on her lips, she underwent a radiotherapy course that included 30 Gy doses in 15 fractions using a 1 cm bolus. Although her lips were initially better after radiation therapy was completed in July 2019, by October 2019, it was documented that the lesions on her lips began to worsen. She also tried light box therapy, ultraviolet light therapy, and gemcitabine chemotherapy without improvement of her symptoms. Due to the worsening paresthesia and increasing tumor size that was not controlled by any other standard treatment, she was directed to our clinic for consultation.

**Figure 1:**
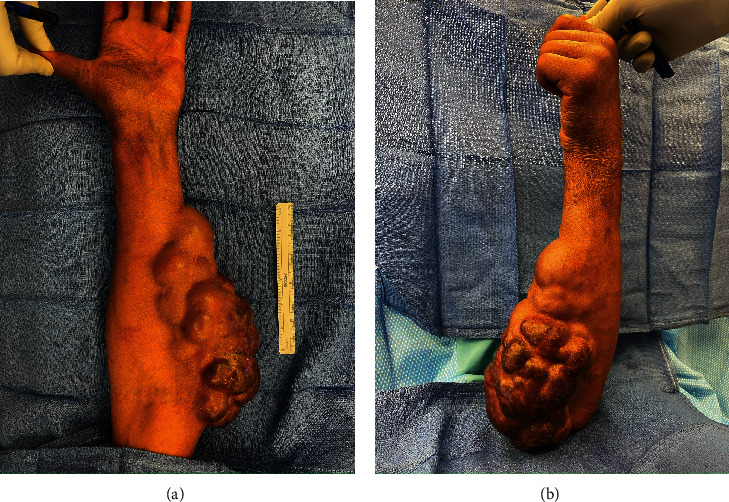
(a, b) Preoperative photos showing LUE mass [7].

When the patient initially presented to our clinic in February of 2023, she had a lobulated, nontender mass that measured 21.0 cm by 10.0 cm covering a significant portion of the left arm overlying the volar aspect of the forearm and extending proximally to the antecubital fossa as seen in Figures 1(a) and 1(b). She had difficulty extending the middle, ring, and small fingers at all joints. There was palpable tethering of the flexor musculature to the tumor with passive extension of the middle and ring fingers. Finger abduction/adduction and thumb opposition were intact. The patient also complained of paresthesias to both the median and ulnar sensory distribution of the left hand. MRI revealed an enhancing mass within the subcutaneous soft tissues of the left forearm that was compatible with the patient's history of MF. While MRI failed to find an area of significant compression of the ulnar nerve, electromyography (EMG) indicated that there was compression of the ulnar nerve.

The patient's clinical history, physical exam, and imaging were concerning for ulnar nerve compression, as well as invasion of flexor musculature. The patient was counseled appropriately and agreed to a radical resection of the tumor at the LUE with cubital tunnel, carpal tunnel, and Guyon canal release to decrease nerve compression symptoms and decrease the mass effect.

The mass of the LUE was outlined where it was visibly demarcated in the skin at the left elbow and forearm. A longitudinal incision overlying the LUE mass was designed for carpal tunnel release and Guyon's canal release. The proximal mass was incised and dissected first, starting at the medial epicondyle. The cubital tunnel was then decompressed proximally and distally. The ulnar nerve was mobilized in situ. Extensive tumor involvement was observed at several of the cutaneous sensory branches distally; these were cauterized and resected with the tumor specimen.

**Figure 2:**
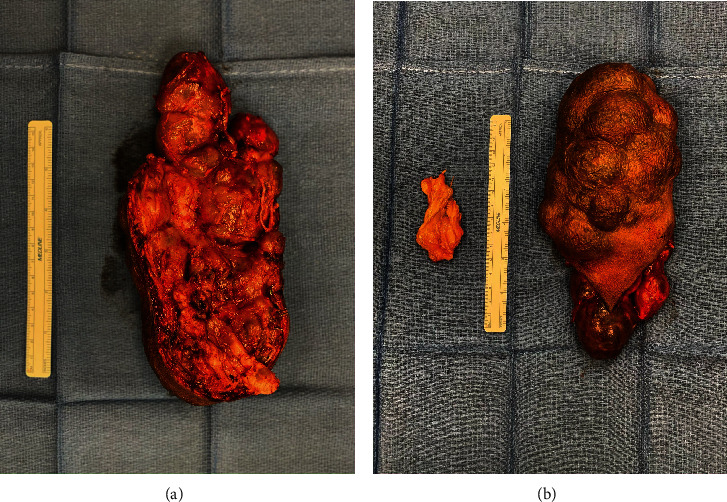
(a, b) Excised masses [8].

The dissection continued distally and into the deep extent of the tumor, involving sharp radical resection of vessels, sensory nerves, flexor carpi ulnaris muscle, and muscle fascia. At several points along the muscle, the nodular tumor extended deep into the flexor mass with no definable plane. Resection was thus unable to continue further in those sites, and debulking was performed with a known positive margin. The resected mass was passed off the table for permanent pathology leaving a 22.0 cm by 11.0 cm defect seen in Figures 2(a) and 2(b).

Attention was then turned to the hand where the superficial sensory and deep motor branches of the ulnar nerve, as well as the median nerve, at the transverse carpal ligament, were decompressed. The remaining hand mass was found to be intimately bound to the flexor tendon sheaths and extended from the distal wrist into the palm within the carpal tunnel. This mass was removed en bloc and measured approximately 7.0 cm by 5.0 cm. The defect at the wrist was closed primarily. The remaining defect of the forearm was closed with a combination of adjacent tissue transfer and split-thickness skin graft.

**Figure 3:**
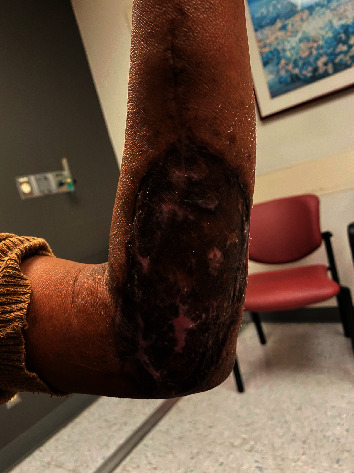
Appearance of arm at 6-week follow-up [9].

A wound vac and bulky soft dressing were applied to the upper extremity. Because of acute blood loss anemia, the patient received two units of packed RBCs on postoperative Day 1 and remained in the hospital until the wound vac was removed from the LUE on postoperative Day 5. At her 2-week follow-up, the patient reported subjective relief of the tingling and improved sensation in her ulnar distribution. She also had improvement in finger extension range of motion. The appearance of her arm at her 6-week follow-up appointment is seen in Figure 3.

## 3. Discussion

Currently, no treatments are considered curative for MF, and management of the disease is largely directed at symptomatic relief through medical treatments. Topical therapies include high-potency corticosteroids and emollients for smaller patches and UV phototherapy for larger patches. Low-dose radiotherapy can be used for refractory small patches. Total skin electron beam radiotherapy can be used for widespread plaques; this treatment modality minimizes the risk of radiotoxicity [10]. When topical therapies are unsuccessful, a range of systemic therapies such as interferon alfa, bexarotene (a retinoic acid derivative), and alemtuzumab can be used. For advanced cases of MF, chemotherapeutic agents such as doxorubicin, gemcitabine, and chlorambucil may be considered [6]. There are few cases of surgical management of MF in the current literature, and currently, there are no recommendations regarding margins for excision to reduce local recurrence [11].

This case demonstrates that surgical management should be considered as an adjunctive treatment option to relieve symptoms secondary to refractory MF. Patients should not be dogmatically denied surgical treatment but instead, be evaluated on a case-by-case basis to determine if surgical management is appropriate. In this case, the patient elected for surgical management of her MF for multiple reasons. First, her MF had already shown itself to be refractory to a number of first-line treatment modalities including chemotherapy, radiotherapy, and UV therapy. Second, physical exam and EMG indicated that the mass was compressing the ulnar nerve. Because the mass was so developed, surgical excision was the only option to remove the source of the compression. Third, the patient was experiencing debilitating symptoms from the mass effect of the tumor. Through shared decision-making, we used surgical excision to decrease the mass effect and decompress the median and ulnar nerves, which gave the patient significant symptomatic relief. She was so satisfied with her results that she requested we evaluate the mass on her right arm. This case presents evidence for consideration of surgical intervention for cutaneous T-cell lymphomatous masses that are refractory to medical treatment and cause debilitating sensorimotor deficits from mass effect.

## Data Availability

The study was a case report, and the data came from chart review. No publicly archived datasets were used for the case report.
